# Comparing Kriging Estimators Using Weather Station Data and Local Greenhouse Sensors

**DOI:** 10.3390/s21051853

**Published:** 2021-03-06

**Authors:** Pei-Fen Kuo, Tzu-En Huang, I Gede Brawiswa Putra

**Affiliations:** Geomatics Department, National Cheng Kung University, Tainan 701, Taiwan; neil860515@gmail.com (T.-E.H.); brawiswa@gmail.com (I.G.B.P.)

**Keywords:** agriculture, oncidium, weather conditions, sensors, kriging

## Abstract

In order to minimize the impacts of climate change on various crops, farmers must learn to monitor environmental conditions accurately and effectively, especially for plants that are particularly sensitive to the weather. On-site sensors and weather stations are two common methods for collecting data and observing weather conditions. Although sensors are capable of collecting accurate weather information on-site, they can be costly and time-consuming to install and maintain. An alternative is to use the online weather stations, which are usually government-owned and free to the public; however, their accuracy is questionable because they are frequently located far from the farmers’ greenhouses. Therefore, we compared the accuracy of kriging estimators using the weather station data (collected by the Central Weather Bureau) to local sensors located in the greenhouse. The spatio-temporal kriging method was used to interpolate temperature data. The real value at the central point of the greenhouse was used for comparison. According to our results, the accuracy of the weather station estimator was slightly lower than that of the local sensor estimator. Farmers can obtain accurate estimators of environmental data by using on-site sensors; however, if they are unavailable, using a nearby weather station estimator is also acceptable.

## 1. Introduction

Weather conditions are the primary factors that have always affected plant growth and agricultural development. However, in recent years, the impacts of climate change have been obvious, such as increased temperatures, highly variable and shifting precipitation patterns, reduced snowpack on mountains, and increased frequency and intensity of extreme weather [1]. Numerous studies have shown that climate change has affected the development of plant crops. For example, in Nepal, the food supply has been affected by increases in CO2 and temperature, and unstable precipitation [2]. Overall, among mountains (35%), hills (42%), and terai (23%) areas in Nepal, the crops such as rice, wheat, and maze in the Terai area (south of the outer foothills of the Himalayas) have decreased in the past decades. Similarly, in California, these abnormal weather patterns impact on agriculture in many ways, including increased pest and disease pressure and crop yield decline. Due to those reasons, climate change brings a negative impact on the agricultural industry [1]. Therefore, in order to come up with an effective strategy for optimizing crop performances under these abnormal weathers, monitoring these weather conditions is critical. 

As previously mentioned, installing on-site sensors and getting data from weather stations are two common methods to collect/observe these weather conditions. Although the sensor could be installed on-site in order to observe accurate weather information, the installation and its management are time-consuming and costly. As an alternative option, the government weather station data could be used in this case, since it is open to the public for free. However, their accuracy is questionable because the locations of these stations are far from the greenhouse. Therefore, in this study two different data sets were used to compare the accuracy of kriging estimators: First, multiple sensors were installed and dispersed throughout an Oncidium greenhouse in order to collect the actual/real local weather data. In addition, this study also utilized the online weather station data from the open data website Central Weather Bureau. The estimators were compared using these two datasets. The data were analyzed over a period of several months in order to determine how seasonal changes affect our study results. If both types of estimators yield similar numbers, farmers with limited budgets can use weather station data to monitor temperature instead of installing their own onsite sensors. Also, researchers will be able to apply historical data from weather stations to represent past weather conditions and use them for time series analysis.

## 2. Literature Review

Environmental factors have a significant impact on agriculture on a global scale. For this reason, it is essential to understand the environmental conditions which affect crop production. In addition, this information will help researchers predict and control dangerous conditions (such as air pollution) [3]. Therefore, determining the accuracy of weather information in a certain location is vital to the health of crops and to the livelihoods of farmers. The most direct way to accomplish this goal is to install the sensors and collect the necessary data. However, due to a limited budget and time, the farmers were unable to install the sensors seamlessly cover the whole study area [4]. An alternative is to use spatial interpolation methods to obtain weather information at a particular place where it can’t be attained directly. For example, several traditional interpolation methods have been commonly used to transform weather point data to spatial distribution, such as the inverse distance weighting method (IDW) and the kriging method. The IDW method is relatively straightforward to compute, but the spatial variation of relationships between points cannot be explored and the accuracy of the estimation is limited [5]. In comparison, to estimate the structure of spatial variance, the kriging approach uses variogram analysis and takes into account spatial autocorrelation [6,7]. Recently, some researchers have focused on the accuracy and the performance of these spatial interpolation methods. For example, Oktavia et al. [4] and Zhang et al. [7] used IDW and kriging spatial interpolation for thermal monitoring of a data center and evaluated the accuracy of these two methods based on the Root Mean Squared Error (RMSE) value. Their results showed that the kriging method performed better.

Kriging, which has been widely used for spatial interpolation [8], has the key advantage of incorporating spatial correlation data, leading to a high accuracy rate. Kriging itself is a category of stochastic interpolation methods that include ordinary kriging, simple kriging, co-kriging, universal kriging, regression kriging, and residual kriging [9]. These approaches have been used in a number of researches. For instance, Wu et al. [10] used the residual kriging approach with input variables of longitude, latitude, and elevation to approximate the average monthly temperature in the United States. Liang et al. [11] used the co-kriging method to model the nitrate-nitrogen daily concentration in an agricultural river. Based on the application in the previous study, kriging was a suitable method for our study since our target predictive variable was the temperature, which has spatial variance [12]. 

Several researchers who have used spatio-temporal kriging to interpolate temperature in the space-time field discovered that it is more accurate than spatial kriging [13]. The additional advantage of spatio-temporal kriging resides in the model’s versatility. This method not only interpolates at unobserved spatial locations but also at unobserved instances of time [14]. This reason makes spatio-temporal kriging an appropriate method to cover the gaps of a time series model not only dependent on the time series, but also including all of its spatial neighbors. In sum, the purpose of this study was to compare the spatio-temporal kriging result by using two different datasets in two different seasons.

## 3. Data and Methods

### 3.1. Study Area & Study Data

**Figure 1:**
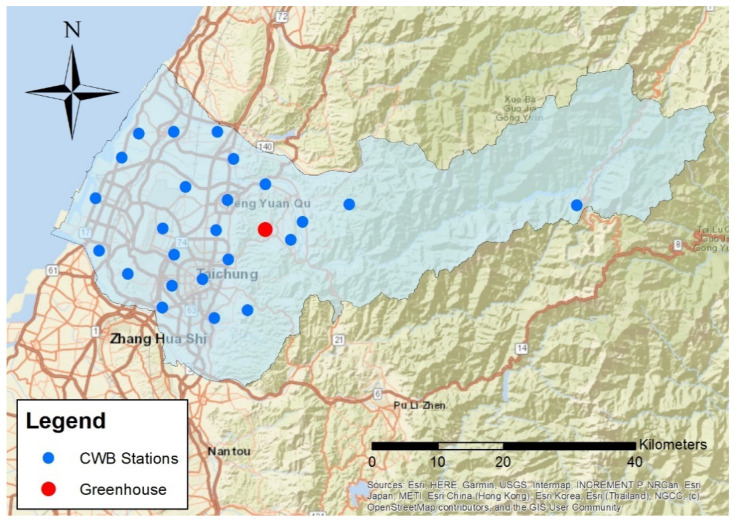
The locations of weather stations.

In this study, the temperature data collected from the sensors and the weather stations from the Central Weather Bureau (CWB) were utilized for the spatio-temporal kriging (http://e-service.cwb.gov.tw (accessed on 5 March 2021)). There were 24 weather stations, the locations of which are indicated by the blue points in Figure 1. The study area was located in the Xinshe District in Taichung City, indicated by the red point. The distance between the greenhouse and the nearest weather station is approximately four km while the farthest weather station is located 48 km from the greenhouse.

The weather stations are able to observe several weather parameters such as station pressure, sea surface pressure, temperature, dew point temperature and relative humidity, wind speed, wind direction, visibility, and cloud amount. Table 1 is an example of one-day data obtained from weather stations. The first column is station name, the date column is the record day, the ObsTime column is the hour of record time, StnPres column is the station average air pressure, Temperature is the average temperature, T.max column is the maximum temperature, T.min column is the minimum temperature, RH column is the average relative humidity, WS column is the average wind speed, precp column is the average precipitation, and sunshine is the light intensity. The weather station provides several weather conditions, such as temperature in degrees Celsius, station air pressure in hPa, relative humidity in percentage, wind speed in m/s, precipitation in mm, and light intensity. 

However, in this study, only temperature data were used for the analysis. Temperature data from the weather stations were downloaded from the CWB Observation Data Inquiry System online. As previously mentioned, the study periods were from 14–20 June 2020 and 14–20 September 2020. These study periods were chosen by considering the sensor performance in two different seasons (Summer and Autumn) and that the observation days with no missing data fell within the study period. On the other hand, the sensors themselves were installed at the end of March 2020 and became fully functional in May 2020. Therefore, this study only covers two seasons during the study period. The raw data from the website is updated hourly, so it was collected 24 times per day at each station within the fourteen days. The temperature value data was also collected from the 44 sensors installed in the greenhouse. The sensors measure the temperature every five minutes, so data was collected 288 times by each sensor per day.

**Figure 2:**
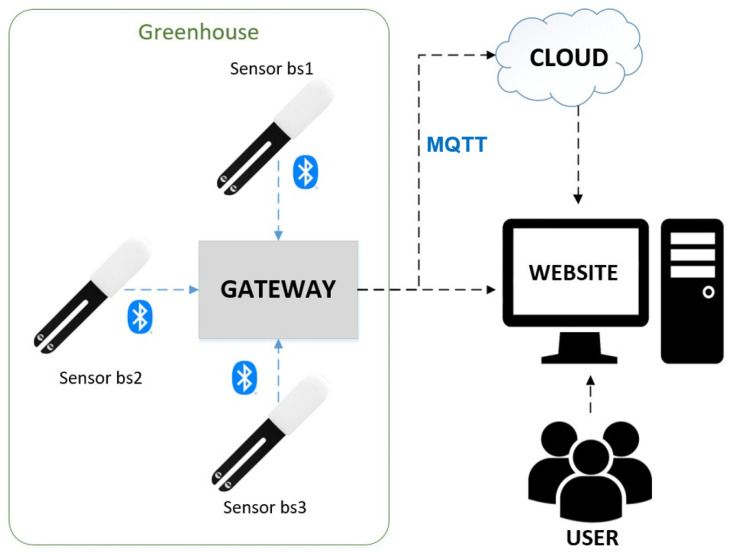
Greenhouse environment monitoring system structure.

**Figure 3:**
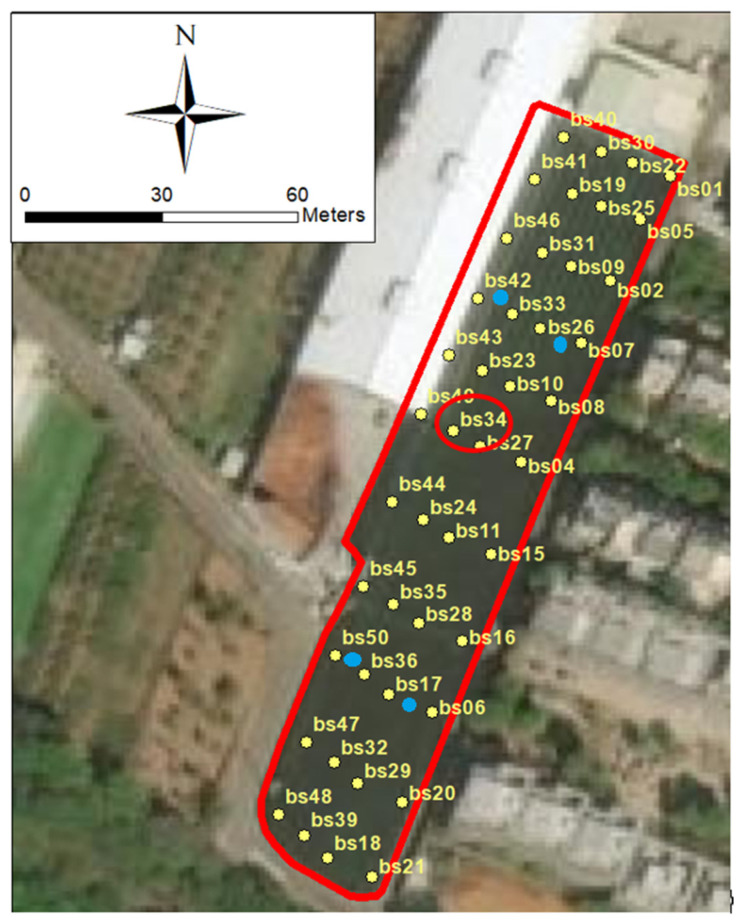
Distribution of sensors and gateways in the Oncidium greenhouse.

The system consists mainly of three main parts: user interface, gateway, and sensor nodes. Figure 2 shows the system structure that illustrates how the environment monitoring system for the greenhouse operates. The sensors, each of which are numbered, are uniformly distributed and deployed throughout the greenhouse to collect environmental information such as temperature, luminosity, and humidity. The data collected by the sensor nodes are transmitted to the gateways via Bluetooth. This study follows the concept of a star network topology where the gateways are deployed in the center of the greenhouse and used to collect the data from sensor nodes, store the data, process, and integrate data [15]. In addition, the gateways are connected to a WiFi network and use a message queuing telemetry transport (MQTT) protocol to transfer data from the gateway to the cloud. The user interface, which is a cloud-based web application, allows users to monitor the environmental data measured by each sensor node in real-time. Figure 3 illustrates the greenhouse with an area of approximately $5400\;m^{2}$ (=180 m×30 m) and the sensor’s deployment location.

**Figure 4:**
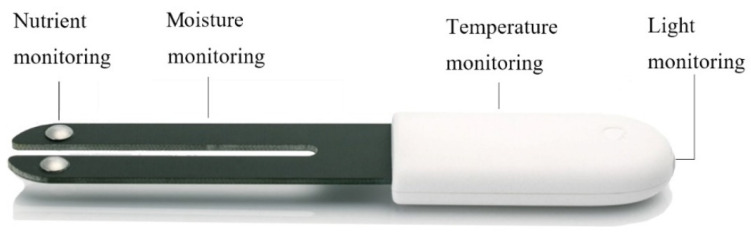
Xiaomi sensor.

**Figure 5:**
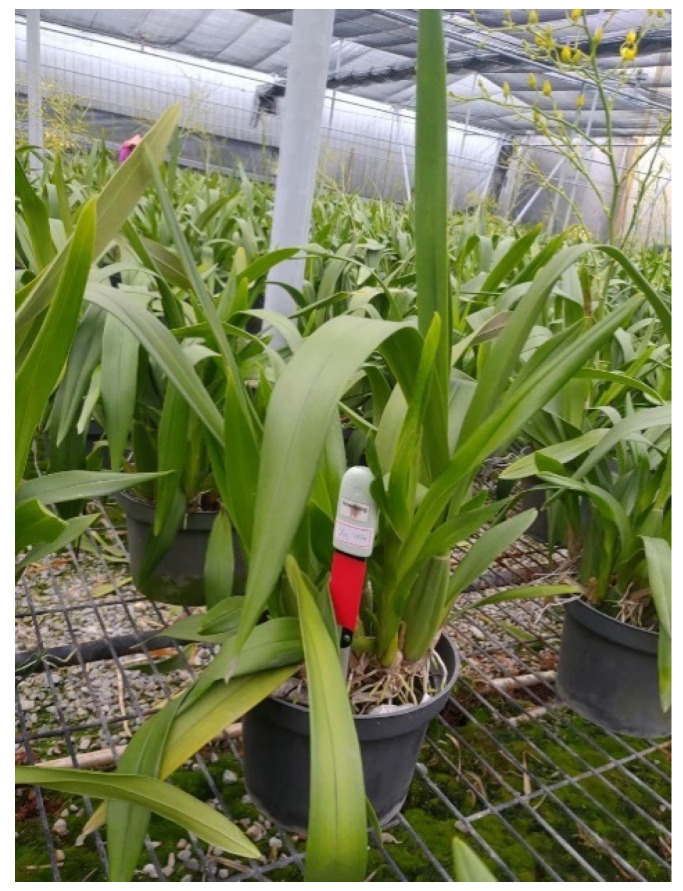
Sensor installation placement.

Bluetooth wireless sensors (shown in Figure 4) from the Xiaomi Corporation were installed throughout the greenhouse (Figure 5) to measure environmental information such as soil moisture and conductivity, as well as light and temperature. This brand was chosen due to its cost-effectiveness compared to other sensors, reliability, and accuracy of up to 0.5 °C. Please see https://www.mi.com/flowermonitor (accessed on 5 March 2021) for more details regarding these particular sensors. Moreover, this type is compatible with the Xiaomi Smart Home App, which means that it will be more convenient for farmers. The parameters of these sensors are shown in Table 2. Regarding battery life, in normal circumstances the battery life could exceed one year if the measurement data is stored only once every 20 min to the receiver (the default scan interval is 1200 s). However, in order to ensure the consistency of the observations and prevent missing data, regular maintenance such as battery replacement was performed every three months from the time the sensors were fully operational in May 2020. It must be noted that the sensor data was set to be recorded every five minutes to reduce the missing rate, which resulted in shorter battery life.

**Figure 6:**
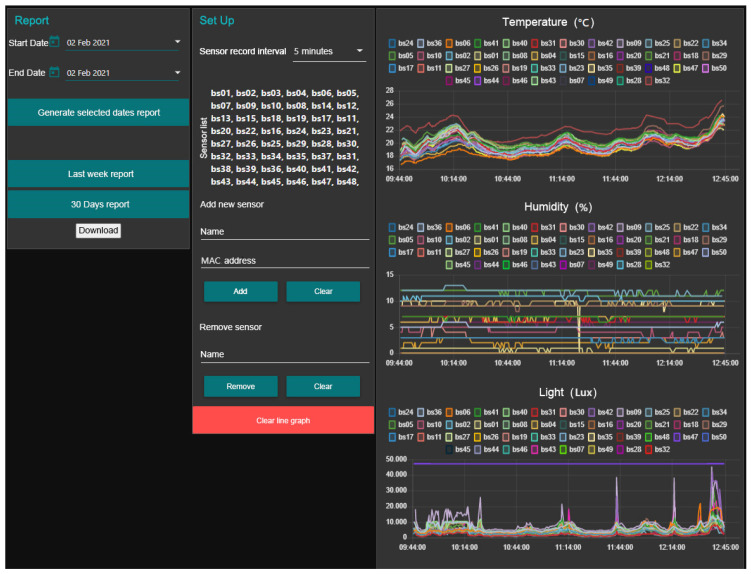
The website that records the sensor data.

The data collected by the sensors were uploaded to a website and downloaded using a computer, the layout of which is shown in Figure 6. For example, real-time environmental data including temperature, soil moisture, and luminosity is displayed from top to bottom. Each collection sensor is assigned its own color, which makes it easier to determine if a particular sensor is not functioning correctly. The middle panel of the platform is where the management and setting interfaces are located, to which sensors can be added or removed and where the time intervals for recording the environmental components can be set. As previously stated, for this study, the intervals were set to five minutes. The sensor data for a specific date can be downloaded on the left panel. The white section at the bottom is for downloading, and the start and end dates are displayed in the blank section at the upper left corner of the screen. 

The measurements of sensor “bs34” were chosen as the real values at the center of the greenhouse, and the temperature data from the other 43 sensors were selected as the spatio-temporal kriging estimator. The reasons for choosing bs34 as the reference for the actual temperature value are because it is located at the centroid of the greenhouse which is far enough away from factors that might affect the temperature, such as the shadows of nearby buildings, and this sensor had no missing data during the study period. Another estimator was based on the 24 weather stations. Thus, the temperature values measured by sensor “bs34” on June 14th—20th and September 14th—20th were the true values that were used to estimate the error by comparing the results obtained from different input data. The dataset itself was filtered which resulted in fewer than 10% missing observations and outliers. The descriptive statistics of the raw data are shown in Table 3. 

### 3.2. Spatio-Temporal Kriging

Kriging is a common method for obtaining unbiased estimators in a particular location for spatial interpolation. Ordinary kriging is the most commonly used kriging method in different subjects. The basic concept of the calculation of it is shown as Equation (1), where Z(x) represents the estimator at the point x, $\omega_{i}$ represents the weight of each sample point, and *n* means the number of the sample point.

(1) $$Z\left(x\right)=\overset{n}{\underset{i=1}{\sum }}\omega_{i}Z\left(x_{i}\right)$$

To determine whether the estimation is unbiased, the weight sum $\left(\omega_{i}\right)$ should equal to 1, as shown in Equation (2).

(2) $$\overset{n}{\underset{i=1}{\sum }}\omega_{i}=1$$

Ordinary kriging interpolation considers the 2-D distance between sample points, also called known/observed points. The coordinates and the Z-values of the known points are used to calculate the semi-variogram for obtaining the weight, which is used to predict the unknown point. The calculation of the variogram can be written as Equation (3), where $\hat{\gamma }\left(h\right)$ represents the semi-variogram, N(h) is the number of the pairs of sample point which separated by distance *h* (Euclidean Distance),$\;Z\left(s_{i}\right)$ and $Z\left(s_{j}\right)$ is the estimator at the coordinate $s_{i}$ and $s_{j}$. Then, the semi-variogram is fitted and can be used to calculate the weight for getting an unknown value of a certain location.

(3) $$\hat{\gamma }\left(h\right)=\frac{1}{2N\left(h\right)}\underset{N\left(h\right)}{\sum }{\left\{\left[Z\left(s_{i}\right)-Z\left(s_{j}\right)\right]\right\}}^{2}$$

**Figure 7:**
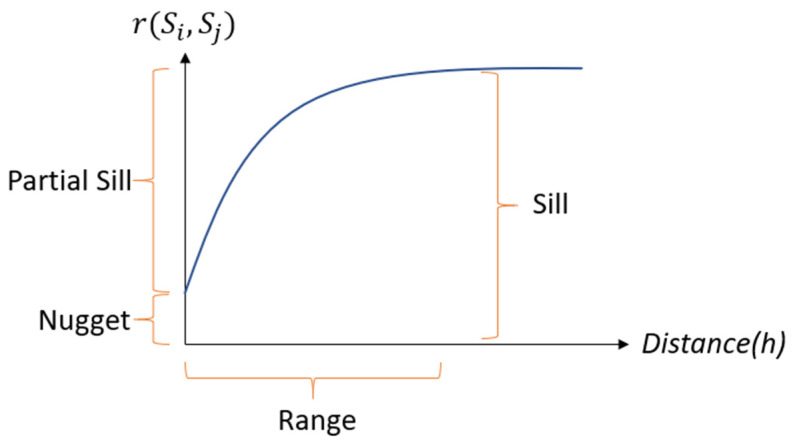
The illustration of semi-variogram.

The illustration of the semi-variogram is shown in Figure 7. The x-axis in Figure 7 represents the distance *h*, the y-axis is the semi-variance, and it shows that the model levels out at a certain distance. The range is the distance where the model first flattens, the value at which the semi-variogram model attains the range (the value on the y-axis) is called the sill, the nugget is the intercept on the y-axis when the distance equal to 0, and the partial sill is the sill minus the nugget. According to McBratney and Webster [16], the semi-variogram value will be 0 when the separation distance is also equal to zero. However, at an extremely small distance of separation, the semi-variogram usually shows a nugget effect higher than 0. The nugget effect could be attributed to spatial variance or measurement errors at distances shorter than the sample interval. Measurement error occurs due to an error underlying in the measurement instrument. Natural phenomena could still vary spatially across a variety of scales. Microscale variations smaller than the sample interval will appear as part of the nugget effect.

In this study, a method known as spatio-temporal kriging was used to spatially interpolate the temperature data over time. This method takes time into account during the kriging process by adapting the covariance function. Unlike the standard 2-D kriging technique, spatial-temporal kriging not only considers the coordinates but also the element of time [14]. Specifically, for each point $s_{i}$, there is a time $t_{i}$ associated with it to calculate the variance between this point and another point of interest with a spatial separation of h as well as their temporal separation u. Thus, the spatio-temporal variogram can be calculated using Equation (4).

(4) $$\hat{\gamma }\left(h,u\right)=\frac{1}{2N\left(h.u\right)}\underset{N\left(h,u\right)}{\sum }{\left\{\left[Z\left(s_{i},t_{i}\right)-Z\left(s_{j},t_{j}\right)\right]\right\}}^{2}$$

After utilizing Equation (4), the weight of each point with a known value can be calculated using the spatio-temporal variogram and the unknown value of a certain location can be determined via interpolation.

For this study, the “sp”, “gstat”, “spacetime” packages for use with software “Rstudio” were selected to process the spatial-temporal kriging. First, the spatio-temporal variogram was constructed based on the sample data collected for predictions, which is similar to a normal 2-D kriging experiment. The simple sum metric model was chosen as the most effective for fitting the model to our variogram. It combines the spatial, temporal and joint nugget effects to restrict the spatial, temporal, and joint variograms into a nugget-free model. Thus, a single spatio-temporal nugget and the variogram can be written as Equation (5) [14].

(5) $$\gamma \left(h,u\right)=nug\cdot 1_{h>0\vee u>0}+\gamma_{s}\left(h\right)+\gamma_{t}\left(u\right)+\gamma_{joint}\left(\sqrt{h^{2}+{\left(k\cdot u\right)}^{2}}\right)$$

where γ(h,u) represents the spatio-temporal variogram, $\gamma_{s}\left(h\right)$ symbolizes the spatial variogram, $\gamma_{t}\left(u\right)$ is the temporal variogram, $\gamma_{joint}\left(\sqrt{h^{2}+{\left(k\cdot u\right)}^{2}}\right)$ represents the joint variogram, and k denotes the spatio-temporal anisotropy scaling parameter. The selected variogram fitting model was used to calculate the weight of the location with a known value for prediction, which allowed for the interpolation of that particular location.

The ten-fold cross-validation technique was utilized to evaluate the kriging method’s performance [17]. This method is one of the cross-validation methods that is commonly used in order to assess the quality of kriging prediction. The sensor data was divided into groups of ten with approximately the same sensor numbers. Then, one data group was set aside as the testing dataset while the remaining nine were utilized as the training datasets. The kriging test was conducted using the training dataset to predict the values of the test dataset. This process was repeated until predictions were determined for all groups. This is primarily used to assess how accurate the kriging model would be in reality. In order to test the prediction accuracy of the kriging, the R-square and RMSE measurements were determined by comparing the prediction values to the observations.

### 3.3. Data Preprocessing

The weather station data was measured automatically per hour, and the sensor data was calculated every five minutes. As mentioned previously, the goal of the study was to compare the accuracy of the spatio-temporal kriging results using these two types of data. Therefore, all the sensor data was converted into the same format for comparison. In order to convert the sensor data into a per-hour format, the average hourly temperature data of each sensor was calculated. With regard to the missing data, the null value was replaced with each individual average.

### 3.4. Comparison

To compare the accuracy of the spatio-temporal kriging predictions using weather station data and sensor data, in this study the Root Mean Square Error (*RMSE*) and Mean Absolute Error (*MAE*) values were compared. Then, the real and predicted temperatures from the weather station and sensor data could also be projected and analyzed as to whether they were affected by the different seasons. Equation (6) was used to calculate the RMSE, and Equation (7) calculates the MAE.

(6) $$RMSE=\sqrt{\frac{1}{n}\overset{n}{\underset{i=1}{\sum }}{\left(y_{i}-\hat{y_{i}}\right)}^{2}}$$

(7) $$MAE=\frac{1}{n}\overset{n}{\underset{i=1}{\sum }}\left|\left(y_{i}-\hat{y_{i}}\right)\right|$$

where n represents the number of samples, $y_{i}$ is the true value (the temperature value measured by sensor bs34), and $\hat{y_{i}}$ symbolizes the predicted value (the interpolation result of spatio-temporal kriging based on the weather station and sensor data). The smaller the value, the more accurate the predicted result.

## 4. Results

### 4.1. Cross-Validation Results

**Figure 8:**
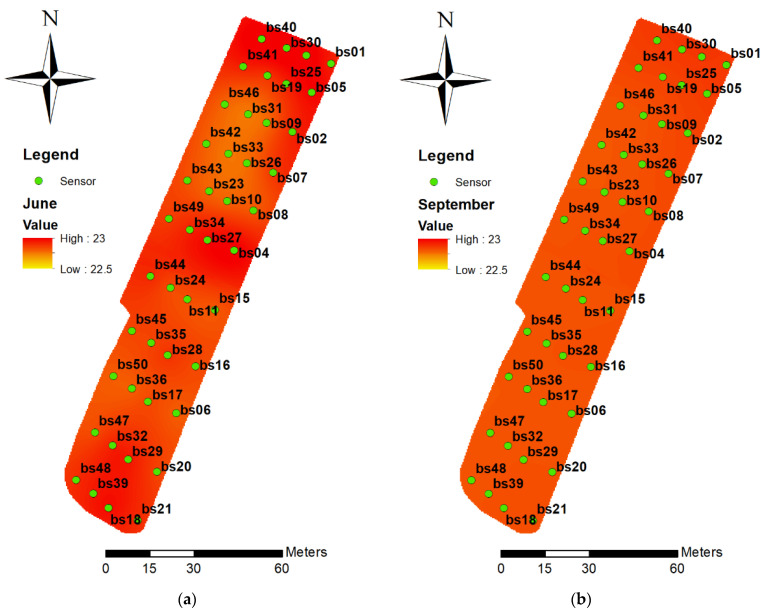
The kriging results for the greenhouse temperatures (**a**) June 14th from 12:00 a.m.—00:55 a.m. (summer); (**b**) September 14th from 12:00 a.m.—00:55 a.m. (autumn).

The kriging results illustrate the temperature variations in the greenhouse that were recorded using sensors, as shown in Figure 8. The average temperatures on July 14th from 12:00 a.m.—00:55 a.m. (Figure 8a) and on September 14th from 12:00 a.m. —00:55 a.m. (Figure 8b) were utilized as the sample, which as shown in the legend, and measured in degrees Celsius. As seen in Figure 7, the average temperatures in July, especially in the northern, middle, and southern sections of the greenhouse, were slightly higher than those in September, which was expected. Furthermore, there were fewer variances in September than in June, largely because there were more missing data on September 14th than on June 14th. Since in this study the missing data were filled in with the averages of all sensor measurements, the temperature distribution, shown in Figure 8b, was less than that in Figure 8a.

**Figure 9:**
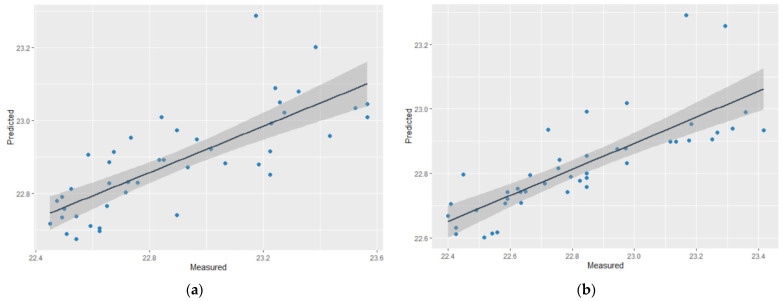
Sample of scatterplots of the observed and predicted greenhouse temperatures (**a**) June 14th from 12:00 a.m.—00:55 a.m. (summer); (**b**) September 14th from 12:00 a.m.—00:55 a.m. (autumn)

Validation is crucial for evaluating a model’s robustness and performance. For this study, the R-square and RMSE values from the results of the k-fold cross-validation were utilized as the parameters to evaluate the kriging from the sensor observations [17]. Figure 9 shows scatterplots of the observed and predicted temperatures (based on 95% prediction intervals). The kriging consistently performed well in both seasons with R-squares of 0.59 and 0.62 (RMSE 0.3, and 0.19) in summer (Figure 9a) and autumn, respectively (Figure 9b).

### 4.2. Comparison Result

To estimate the temperature at the center of the greenhouse (sensor # bs34) on June 14th—20th and September 14th—20th, the temperature data from the nearby weather stations and 43 greenhouse sensors were used to determine the spatio-temporal kriging respectively. The temperature value, measured by sensor bs34, was set as the true value and the difference between the predicted and the true value was the error. To compare the model performance, the RMSE and the MAE were calculated, as shown in Table 4. The temperature value that was measured by sensor bs34 was designated as $y_{i}$ in Equations (6) and (7) and the kriging results from the sensors and the CWB weather station were defined as $\hat{y_{i}}$. The RMSE was calculated by dividing the sum of the square from the difference between each bs34 observation and the kriging result with the number of sample sizes within one week (4032 sample for CWB sensor data; 7392 sample for sensor data). In addition, the results from adding the absolute value to the subtracted true value and the kriging predicted value were divided by the number of sample sizes from a week of data were calculated in order to obtain the MAE value.

**Figure 10:**
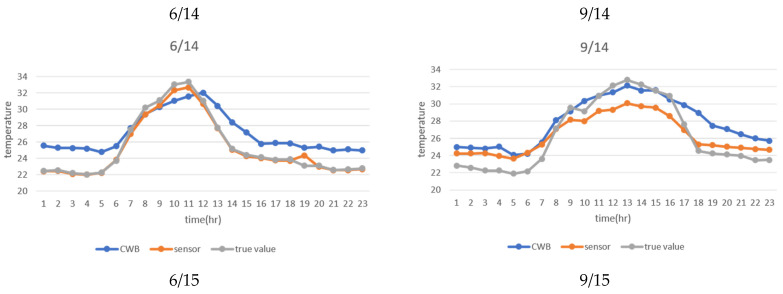

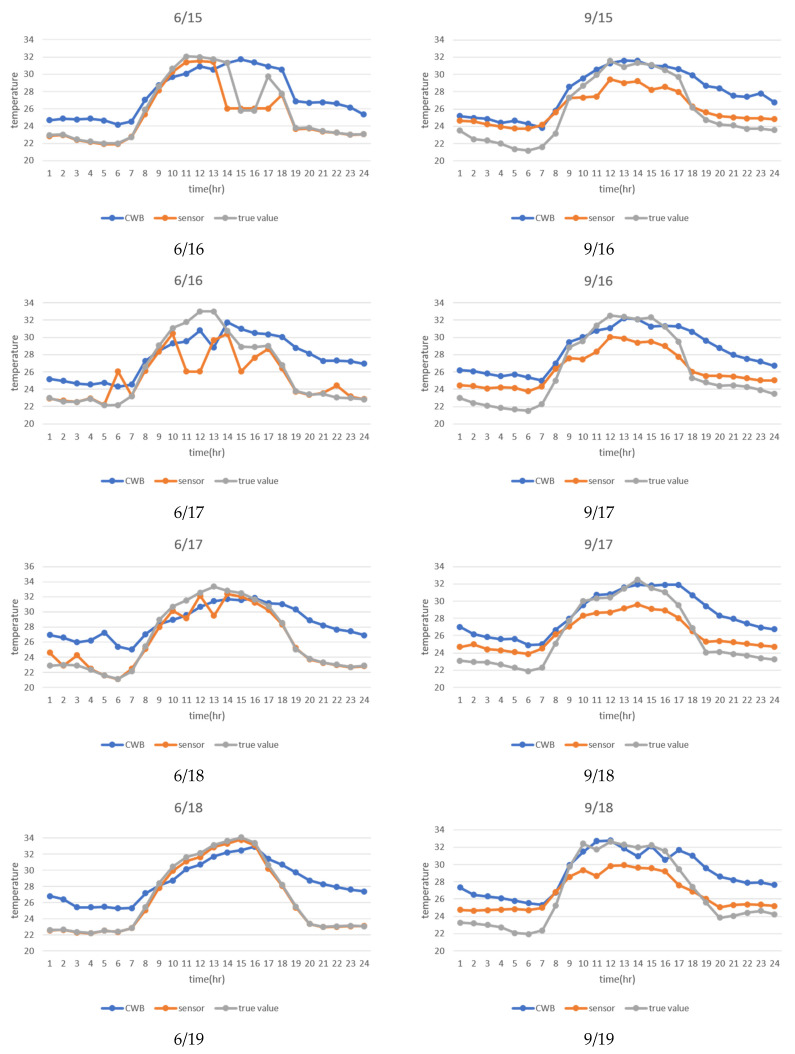

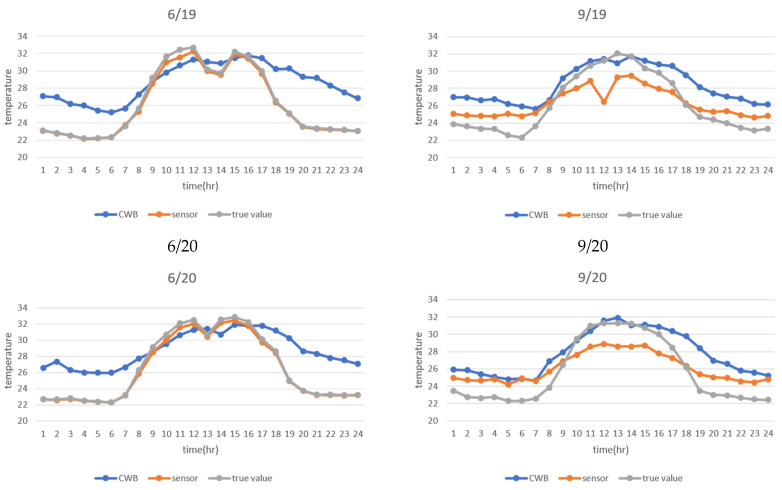
Line chart of the predicted and true values.

In addition, the true value and the two estimators were plotted on the graphs below to compare the differences in the results between June and September. Figure 9 shows the line chart of the true and the predicted values based on the weather station and sensor data. The blue line is the predicted value from the weather station data, the orange line is the predicted value from the sensor data, and the gray line shows the observed value from sensor bs34 (real value). The x-axis is the time, and the y-axis is the temperature. As shown in Figure 10, the sensor data prediction on June 14th—20th was closer to the true value than the one on September 14th—20th. During this week, the weather station data prediction was a bit higher than that from the sensor data.

## 5. Conclusions

According to Table 4, the RMSE and the MAE from the sensor data predictions were all lower than from the weather station data. Thus, the spatio-temporal kriging sensor data results were more accurate than the weather station data. Moreover, the RMSE and MAE values of the sensor data prediction in September were obviously higher than in June. The possible reason for this is that the missing rate (percentage of the data missing in dataset) of the sensor data in September was higher than in June. These results indicate that fewer sample points used for the spatio-temporal kriging will obtain less accurate results, as seen in September.

As shown in Figure 10, the trend of the predicted value from the sensor data and true value were similar on June 14th—20th. The RMSE and MAE were also similar. Moreover, the prediction based on sensor data in June was more accurate than the one in September. On June 14th—20th, the prediction based on weather station data was a bit higher than that from the true value and sensor data, except from 9 a.m. to 12 a.m. As indicated in all figures, most of the predictions from sensor data were lower than those from weather station data. The possible reason is that several fans are installed in the greenhouse and only operated during summer. When the results in summer (June) and autumn (September) were compared, there were no obvious differences between them. Moreover, there was a more significant difference between that from the sensor estimator and the true value at noon (11 a.m. to 12 a.m.) than from the CWB estimators on 6/17 and 9/20. The possible reason is that the daily average was used to fill the missing value at and these two days have more missing values than usual.

According to our results, the local-sensor kriging method performed better than the weather station data. It must be noted that the distance between the known and the unknown point will affect the accuracy of the spatio-temporal kriging result. The sample size and original value of the data integrity will also affect the accuracy of the result. Thus, future scholars may wish to focus on defining the optimal sample size of sensors for the kriging method and analyze the difference between the kriging estimators. Another limitation is the high consumption rate of the sensor battery. When some sensors are out of battery, the missing value increases and then reduces the accuracy of the kriging result. The researchers also suggest future work could include long-term observation of seasonal and yearly temperature.

## Figures and Tables

**Table 1:** Weather station data.

Station	Date	Obs Time	StnPres	Temperature	T.Max	T.Min	RH	WS	Precp	SunShine
467490	6/14/2020	16	1002.2	26.1	34.1	24.4	85	0.6	32	0.0
467770	6/14/2020	9	1008.9	30.7	32.4	27.1	70	4.3	0.0	1.0
C0F000	6/14/2020	4	976.3	24.7	33.0	24.3	100	0.0	0.0	...
C0F850	6/14/2020	11	969.5	30.5	32.4	23.0	61	3.2	0.0	...
C0F861	6/14/2020	23	786.0	16.1	24.5	13.6	100	0.0	0.0	…
C0F930	6/14/2020	01	998.9	28.2	35.1	26.7	94	0.4	0.0	...
C0F970	6/14/2020	17	994.9	27.0	33.6	25.2	90	1.6	0.5	...
C0F9A0	6/14/2020	4	963.2	24.9	34.8	23.4	85	0.5	0.0	...
C0F9I0	6/14/2020	1	988.4	27.1	32.5	25.6	86	1.3	0.0	...
C0F9L0	6/14/2020	18	983.6	26.6	33.1	25.9	99	0.5	13.0	...
C0F9M0	6/14/2020	7	985.3	28.8	33.0	25.8	67	0.6	0.0	...
C0F9N0	6/14/2020	24	1005.9	26.9	35.7	25.7	90	1.4	0.0	...
C0F9O0	6/14/2020	23	994.2	27.8	35.8	27.3	81	0.0	0.0	...
C0F9Q0	6/14/2020	1	992.8	26.4	33.8	25.0	89	1.3	0.0	...
C0F9S0	6/14/2020	2	998.8	27.6	33.9	26.0	91	0.7	0.0	...
C0F9T0	6/14/2020	10	1000	32.1	33.7	26.5	61	1.7	0.0	…
C0F9U0	6/14/2020	10	1004.6	32.7	34.4	26.6	61	2.0	0.0	...
C0F9V0	6/14/2020	6	954.2	25.2	32.6	23.5	82	1.0	0.0	...

**Table 2:** Parameters of the Xiaomi sensors.

Size	120.5 mm × 24.5 mm × 12.5 mm
Wireless Connection	Bluetooth 4.1 BLE
Operating Voltage	3 V
Battery	CR2032 button cell battery

**Table 3:** Statistical descriptions of the raw data for temperatures.

	June 14th—20th			September 14th—20th		
	CWB	Sensor	bs34	CWB	Sensor	bs34
Min.	12.6	20.59	21.12	12.1	20.6	21.19
Q1	26.6	22.76	22.85	26.5	23	22.98
Median	28.6	23.93	23.72	28.3	24.51	24.12
Mean	28.23	26.04	26.05	28.11	26.314	25.86
Q3	30.8	30.00	30.16	30.5	30.31	29.57
Max.	35.7	37.81	34.06	35.7	35.03	32.77
Stdv	3.68	3.73	3.83	3.79	2.85	3.21

Unit: degrees Celsius.

**Table 4:** RMSE and MAE (hourly temperatures).

	RMSE		MAE	
Date	CWB	Sensors	CWB	Sensors
June 14th—20th	3.01	1.10	2.63	0.47
September 14th—20th	2.66	1.87	2.26	1.72

## Data Availability

Not applicable.
